# P2X4 receptors on myeloid cells mediate tissue injury in a murine model of trauma and hemorrhagic shock

**DOI:** 10.21203/rs.3.rs-8196425/v1

**Published:** 2025-11-28

**Authors:** Taha Kelestemur, Zoltan H. Nemeth, Pal Pacher, H. Thomas Lee, Mihwa Kim, Mehmet S Aydin, Ugur Akcan, György Haskó

**Affiliations:** Department of Anesthesiology, Columbia University, New York, NY, USA; Department of Surgery, Morristown Medical Center, Morristown, NJ, USA; Laboratory of Cardiovascular Physiology and Tissue Injury, National Institute on Alcohol Abuse and Alcoholism, National Institutes of Health, Bethesda, MD, USA; Department of Anesthesiology, Columbia University, New York, NY, USA; Department of Anesthesiology, Columbia University, New York, NY, USA; Department of Histology & Embryology, Faculty of Medicine, Istanbul Medipol University, Istanbul, Turkiye; Department of Neurology, Columbia University, New York, NY, USA; Department of Anesthesiology, Columbia University, New York, NY, USA

**Keywords:** purinergic signaling, P2X4, P2X7, trauma hemorrhagic shock

## Abstract

Trauma and hemorrhagic shock (T/HS) trigger systemic inflammation and multiorgan injury, yet the molecular mediators of this response remain incompletely defined. Purinergic receptors, including P2X4 and P2X7, are key regulators of innate immune signaling and may contribute to post-trauma organ dysfunction. Here, we assessed the roles of P2X4 and P2X7 in T/HS-induced injury across multiple organs using genetic and pharmacologic approaches in murine models. Global P2X4 knockout (KO) mice exhibited significantly reduced injury in the lung as reflected by improved histopathology, decreased myeloperoxidase activity, and preserved tissue architecture. Additionally, P2X4 KO reduced liver and kidney injury, as indicated by plasma liver enzymes and blood urea nitrogen levels. Myeloid-specific P2X4 deficiency recapitulated these protective effects, suggesting a central role for myeloid cell–mediated P2X4 signaling in multiorgan injury. Bulk RNA sequencing of lung tissue from P2X4 KO mice revealed altered expression of immune response genes, including downregulation of P2X7. Pharmacological inhibition of P2X7 reduced injury in the lung, liver, and kidney. Both P2X4 and P2X7 expression were downregulated in affected organs following T/HS and in LPS-stimulated macrophages in vitro. These findings identify P2X4, particularly in myeloid cells, as a key driver of multiorgan injury following T/HS, and support further investigation of P2 receptor modulation as a therapeutic strategy in trauma-induced organ injury.

## Introduction

Hemorrhagic shock is a critical condition resulting from significant blood loss, leading to inadequate tissue perfusion and oxygenation. The main cause of hemorrhagic shock is trauma, but it can also result from severe burns, perioperative blood loss, gastrointestinal bleeding, and aneurysm rupture, all of which can cause rapid blood loss (1). The pathophysiology involves decreased circulating blood volume, leading to reduced cardiac output and systemic hypoperfusion. This insufficiency impairs cellular metabolism, causing hypoxia, anaerobic respiration and accumulation of metabolic waste. Consequently, endothelial dysfunction occurs, leading to increased vascular permeability and interstitial edema (2). Once the hemorrhage is controlled and the patient is resuscitated, hemorrhagic shock frequently results in multiple organ failure (MOF). This occurs due to the combined effects of hypovolemic-ischemic damage and the reperfusion injury induced by the resuscitation efforts involving intravenous fluids and blood products (3). The primary pathogenetic factor contributing to MOF is inflammation, which arises from the generation of oxygen free radicals and the release of damage-associated molecular patterns (DAMPs) (4–7). While ischemia-reperfusion is the major trigger behind free radical production, trauma releases additional DAMPs to exacerbate inflammation.

Extracellular ATP has recently emerged as a significant DAMP that is released from the intracellular space into the extracellular space during hypoxia and inflammation (8). The detection of released ATP by P2 purinergic receptors on the surface of immune cells- whether through paracrine, autocrine, or endocrine mechanisms- alerts the immune system to potential danger, initiates inflammation, and orchestrates host immunity. P2 receptors are categorized into two classes: ionotropic P2X (P2X1 to 7) receptors, which conduct small cations such as Ca^2+^, Na^+^, and K^+^, and metabotropic P2Y (P2Y_1_, P2Y_2_, P2Y_4_, P2Y_6_, P2Y_11_, P2Y_12_, P2Y_13_, and P2Y14) receptors, which signal via G protein coupling. ATP has been identified as the first DAMP capable of activating the NLRP3 inflammasome (9, 10)., The NLRP3 inflammasome is a multi-protein complex in innate immune cells that detects cellular stress or danger and triggers inflammation. This occurs by triggering the release of interleukin (IL)-1, IL-18, and IL-33, along with inducing pyroptosis, an inflammatory form of cell death. The inflammasome-activating effect of ATP is mainly mediated by P2X7 and, to a lesser degree, by P2X4 (11, 12). One explanation for the central role of P2X4 and P2X7 receptors in regulating inflammation is that they are expressed at high levels on myeloid inflammatory cells, such as macrophages and dendritic cells (13–17).

We have recently been interested in studying how P2X4 receptors regulate ischemic and inflammatory organ injury. Our findings demonstrate that the role of P2X4 receptors varies depending on the organ injury model used: they had a detrimental effect in ischemic kidney injury (18) but offered protection in sepsis (11). While in kidney injury the deleterious effects of P2X4 receptors were likely mediated by the activation of the NLRP3 inflammasome (18), the protective effects of P2X4 receptors in sepsis were mediated by increased killing of bacteria in macrophages (11). This suggests that it is challenging to predict the role of P2X4 receptors in a complex injurious insult such as trauma and hemorrhagic shock. Thus, in the current study, we have investigated the role of this ancient signaling pathway comprising ATP release and P2X4 receptors in regulating the host’s response to trauma and hemorrhagic shock.

## Materials and Methods

### Animals

All procedures involving mice were conducted with the approval of the Columbia University Institutional Animal Care and Use Committee (IACUC) under approval number AABL4551/2021. Adult male P2X4^+/+^ (C57BL/6) and P2X4^−/−^ mice, along with LysM^Cre^-P2X4^fl/fl^ mice and their wild-type controls P2X4^fl/fl^, aged 8–12 weeks (n = 4 per group), were bred at Charles River (Wilmington, MA, USA)(11). Mice were provided with food and water *ad libitum* and were housed in a room with a 12-hour light-dark cycle under standard pathogen-free conditions.

### Drugs, study design, and induction of traumatic hemorrhagic shock

In one set of studies, mice were randomly allocated into the following groups: trauma/sham shock (T/SS) mice receiving vehicle (saline), T/HS mice receiving vehicle (saline), T/HS mice receiving the P2X4 receptor antagonist 5-BDBD (1 mg/kg) (3579, Tocris, USA), and T/HS mice receiving the P2X7 receptor antagonist A740003 (5 mg/kg) (3701, Tocris, USA). Furthermore, in a separate set of tests, germline P2X4^−/−^ (KO) and myeloid-specific P2X4 deficient (LysMCre-P2X4^fl/fl^) mice, along with their controls P2X4^+/+^ (WT) and P2X4^fl/fl^, respectively, underwent T/SS or T/HS. Mice were given various drugs (5-BDBD or A740003) via intraperitoneal injection 30 minutes before T/SS or T/HS in the pharmacological studies.

The mice were anesthetized with 1% isoflurane, and their rectal temperature was maintained between 36.5 and 37.5°C using a feedback-controlled homeothermic blanket heating system (SS-01, Somno-suite, Kent Scientific). Hemorrhagic shock was induced using a fixed-pressure model, as we have described before (19, 20). A 2 cm midline laparotomy was first performed on sedated mice and then closed with a 4 − 0 silk suture (034902, Covetrus, USA). Catheters were then inserted into the right and left femoral arteries for blood pressure monitoring and blood sampling, respectively. A sterile 1-ml syringe with a 30G needle connected to PE-10 tubing containing 0.2 ml of 1% heparinized saline was used for blood collection, with each mouse receiving 1U of heparin. Blood pressure was monitored with a continuous blood pressure device (ML870, Powerlab 8/30, ADInstruments, Colorado Springs, CO, USA). After a 5-minute baseline blood pressure measurement, mice were given either a drug or a vehicle. Shock was then induced for 2.5 hours, with blood pressure sustained between 28 and 32 mmHg by withdrawing or reinfusing collected blood. At the end of the shock period, the mice were resuscitated with Ringer’s Lactate at three times the volume of lost blood over a duration of 15 minutes. Three hours postresuscitation, the mice were euthanized, and bronchoalveolar lavage fluid (BALF), blood, and tissue samples were collected. T/SS animals received the same treatments as the other animals, except for blood removal.

### Tissue preparation, MPO analysis, lung permeability, and Western blot

#### Tissue preparation, MPO analysis, lung permeability, and Western blot

To assess neutrophil sequestration in the lungs after T/SS or T/HS, myeloperoxidase (MPO) activity was measured using an MPO activity kit (MAK068, Sigma, USA). The supernatant from lung lysates was analyzed following the manufacturer’s instructions. The Evans blue dye (EBD) method was used to evaluate lung permeability. EBD was injected into the tail vein, and about 1 ml of blood was collected from the tail artery 10–20 minutes later. For BALF collection, after making a small incision, a syringe with a 23G needle containing 1 ml of sterile saline was inserted into the trachea, then saline was instilled into the lungs and aspirated back out. The supernatant of BALF was measured spectrophotometrically at 620 nm. The amount of Evans blue dye in the BALF was presented as a ratio relative to its plasma concentration. Lung specimens from four mice in the same group were homogenized in 1X RIPA Buffer (20–188, Millipore Sgima, USA) with a protease/phosphatase inhibitor cocktail (P8340, Sigma, USA). The resulting homogenate was then centrifuged at 13,000 x g for 10 minutes at 4°C. The total protein content was measured with a Qubit 4.0 Fluorometer (Thermo Fisher, USA) following the manufacturer’s instructions. Twenty micrograms of protein per sample were size-fractionated utilizing a 4–20% Mini-Protean TGX Stain-Free electrophoresis gel and then transferred to a PVDF membrane with the Mini Trans-Blot Electrophoretic Transfer System (1703930, Bio-Rad, USA). The membranes were first incubated in a blocking solution of 5% non-fat dried milk in 50 mM Tris-buffered saline containing 0.1% Tween 20 (TBS-T) for 1 hour at room temperature. Subsequently, the membranes were rinsed with 50 mM TBS-T and incubated overnight with rabbit monoclonal anti-P2X4 (APR-024, Abcam, USA) at 1:1000, and rabbit polyclonal anti-P2X7 (APR-008, Abcam, USA) at 1:2000. The following day, the membranes were rinsed with TBS-T and then incubated with a horseradish peroxidase (HRP)-conjugated goat anti-rabbit secondary antibody (ab97051, Abcam, USA) diluted to 1:5000 in the blocking solution for 2 hours at room temperature. To ensure equal protein loading, the membranes were stripped and re-analyzed using an HRP-conjugated anti-ß actin antibody (ab20272, Abcam, USA). The membranes were probed using the Clarity Western ECL Substrate Kit (1,708,280, Bio-Rad, Life Sciences Research, USA) and imaged with ChemiDoc MP imaging system (Bio-Rad, Life Scineces Research, USA)

### RNAseq

Lung samples from 4 WT and 4 KO mice subjected to T/HS were homogenized with 1 ml of Trizol and a 1.5 mm stainless steel bead, using the Qiagen TissueLyser II for 2 minutes at 25 Hz, with the process repeated twice. RNA samples were quantified using Qubit 2.0 Fluorometer (Life Technologies, Carlsbad, CA, USA) and RNA integrity was checked using Agilent TapeStation 4200 (Agilent Technologies, Palo Alto, CA, USA). RNA sequencing libraries were prepared using the NEBNext Ultra RNA Library Prep Kit for Illumina using manufacturer’s instructions (NEB, Ipswich, MA, USA). Briefly, mRNAs were initially enriched with Oligod(T) beads. Enriched mRNAs were fragmented for 15 minutes at 94°C. First strand and second strand cDNA were subsequently synthesized. cDNA fragments were end repaired and adenylated at 3’ends, and universal adapters were ligated to cDNA fragments, followed by index addition and library enrichment by PCR with limited cycles. The sequencing library was validated on the Agilent TapeStation (Agilent Technologies, Palo Alto, CA, USA), and quantified by using Qubit 2.0 Fluorometer (Invitrogen, Carlsbad, CA) as well as by quantitative PCR (KAPA Biosystems, Wilmington, MA, USA). The sequencing libraries were clustered on a flowcell. After clustering, the flowcell was loaded on the Illumina HiSeq instrument (4000 or equivalent) according to manufacturer’s instructions. The samples were sequenced using a 2×150bp Paired End (PE) configuration. Image analysis and base calling were conducted by the Control software. Raw sequence data (.bcl files) generated the sequencer were converted into fastq files and de-multiplexed using Illumina’s bcl2fastq 2.17 software. After investigating the quality of the raw data, sequence reads were trimmed to remove possible adapter sequences and nucleotides with poor quality. The trimmed reads were mapped to the mouse reference genome available on ENSEMBL using the STAR aligner (version 2.7.11b). The STAR aligner is a splice aligner that detects splice junctions and incorporates them to help align the entire read sequences. BAM files were generated as a result of this step. Unique gene hit counts were calculated by using feature Counts from the Subread package v.1.5.2. Only unique reads that fell within exon regions were counted. Heatmaps (version 2.27.0) and volcano plots (version 1.20.0) are created in R using the pheatmap and ggplot packages, respectively. EdgeR (version 4.0.9) is employed to analyze differentially expressed genes based on measured transcripts expressions. Log2 fold change (± 0.25) values and standard error (lfcSE) were calculated. The p-values obtained from the Wald test were corrected using the Benjamini–Hochberg method (padj). Results were deemed statistically significant when padj ≤ 0.05. KEGG pathway and Gene Ontology (GO) enrichment analyses for differentially expressed genes were performed utilizing the R cluster Profiler package.

### Assessment of AST, ALT, and BUN concentrations

Plasma samples were analyzed to measure the concentrations of aspartate aminotransferase (AST), alanine aminotransferase (ALT), and blood urea nitrogen (BUN). The samples were diluted with AST (105135 Abcam, USA), ALT (MAK052 Sigma, USA), and BUN (EIABUN Invitrogen, USA) reagents, respectively, and the resulting light signal was measured using a spectrophotometer.

### Histopathological evaluation of pulmonary injury

Portions of the lung were sectioned at a thickness of 5 μm, stained using the hematoxylin-eosin (H&E) technique, and then scanned with a Leica AT2 slide scanner (Leica, USA). Lung sections were histopathologically assessed based on the following parameters: 1. neutrophils in the alveolar spaces, 2. neutrophils in the interstitial space, 3. hyaline membranes, 4. proteinaceous debris occupying the airspaces, 5. alveolar septal thickening, all conducted by a blind observer (19, 20).

### Culture and treatment of RAW 264.7 macrophages

RAW 264.7 macrophages (ATCC) were cultured in Dulbecco’s modified Eagle’s medium (DMEM) supplemented with 10% (v/v) fetal bovine serum (FBS), 100 μg/ml streptomycin, and 100 U/ml penicillin. The cells were incubated at 37°C in a 5% CO2 atmosphere. For P2X4 and P2X7 expression analysis, the cells were treated with 10 μg/ml LPS (O55:B5 from Sigma) or medium control for 5 hours. At the end of this period, cells were homogenized using TRIzol reagent.

### Real-time (RT)-qPCR

All reagents used in this procedure were obtained from Thermo Fisher – Applied Biosystems, USA. The following primers were used for the PCRs: P2X4 forward: ATCCCTTCTGCCCCATATTC; P2X4 reverse: TAGCCAGGAGACACGTTGTG; P2X7 forward: CCCTGCACAGTGAACGAGTA; P2X7 reverse: AGACAGGTCGGAGAAGTCCA; 18S forward: GCAATTATTCCCCATGGAACG; and 18S reverse: GGCCTCACTAAACCATCCAA.

Snap-frozen samples were homogenized with TRIzol reagent, and RNA was extracted following the manufacturer’s instructions. Then, reverse transcription was performed to generate cDNA. The RT-qPCR procedures, using 20–100 ng of cDNA, were carried out on an Applied Biosystems QuantStudio 3 PCR machine with Master Mix PowerUp and appropriate primers. Data analysis was performed using the 2 − Δ/ΔCT method as previously described, with normalization to the relevant housekeeping genes.

### Statistics

The two-tailed unpaired Student’s t-test was used to compare two groups. One-way ANOVA was used to compare three or more groups. The software used was GraphPad Prism version 8. Results were considered statistically significant when the p-value was less than or equal to 0.05.

## Results

### P2X4 and P2X7 receptors contribute to T/HS-induced lung injury

**Figure 1:**
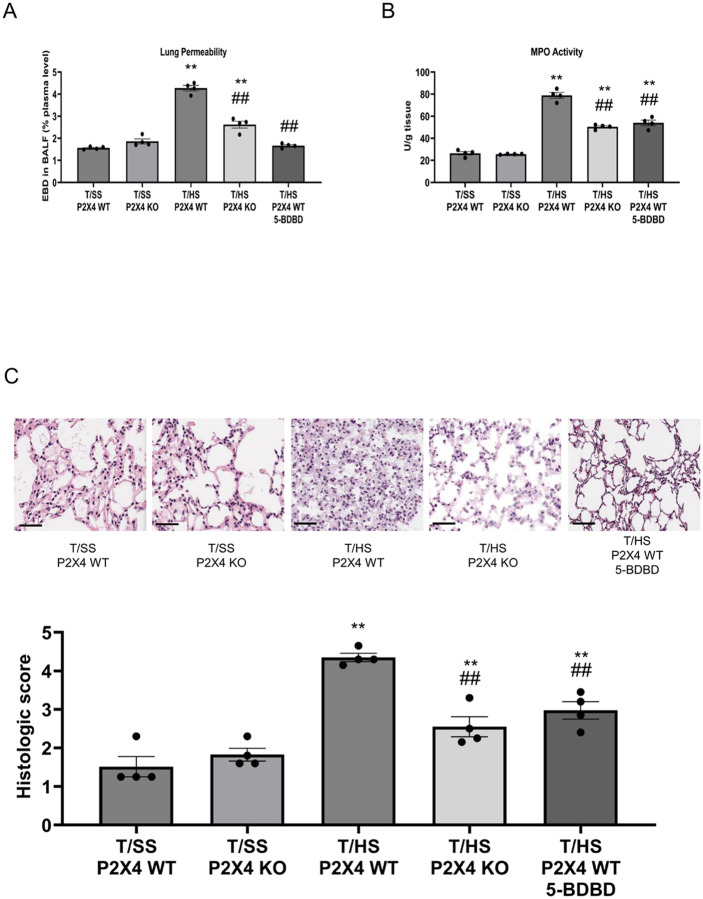
P2X4 regulation of lung permeability and MPO activity. Lung permeability was determined by measuring Evans blue dye (EBD) extravasation (A) and neutrophil sequestration was determined spectrophotometrically by measuring MPO activity (B). Histological lung injury (C) was evaluated using five independent parameters (neutrophils in the alveolar space, neutrophils in the interstitial space, hyaline membranes, proteinaceous debris filling the airspaces, and alveolar septal thickness). Data are mean ± SEM (n = 4/group). **p < 0.01 compared with WT T/SS, ^##^p < 0.01 compared with WT T/HS.

**Figure 2:**
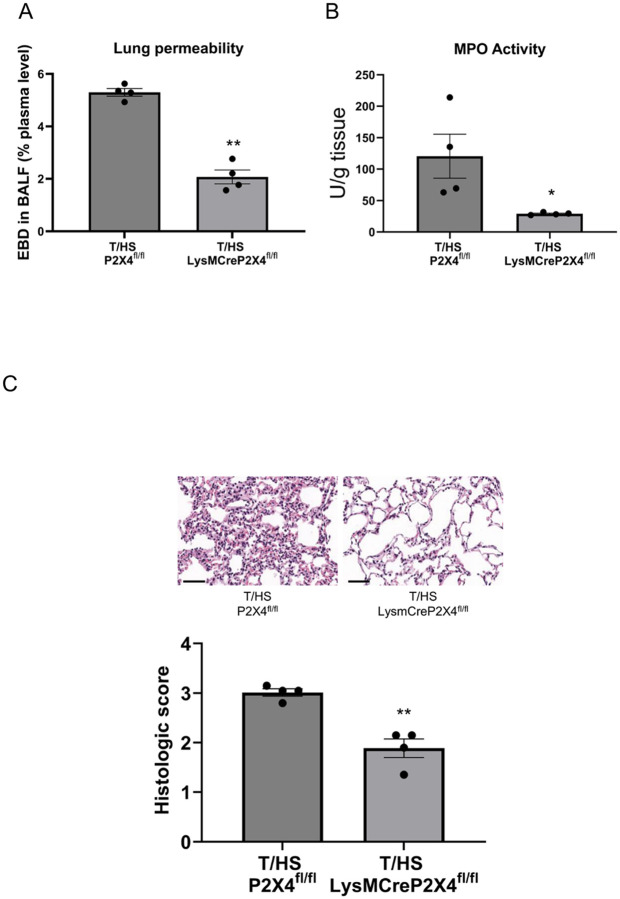
Conditional P2X4 deficiency in myeloid cells protects against lung injury. Lung permeability was determined the using EBD method (A) and neutrophil sequestration was determined spectrophotometrically by measuring MPO activity (B). Histological lung injury (C) was evaluated using five independent parameters (neutrophils in the alveolar space, neutrophils in the interstitial space, hyaline membranes, proteinaceous debris filling the airspaces, and alveolar septal thickness). Data are mean ± SEM (n = 4/group). Data are mean ± SEM (n = 4/group). **p < 0.01 compared with P2X4^fl/fl^ T/HS, *p < 0.05 compared with P2X4^fl/fl^ T/HS.

We evaluated the role of P2X4 and P2X7 receptors in regulating T/HS-induced lung injury. Both P2X4 KO mice and mice pretreated with the P2X4 selective antagonist 5-BDBD exhibited decreased lung permeability (Fig. 1A) and decreased MPO activity (Fig. 1B) compared to WT or vehicle-treated mice, respectively, following T/HS. The lung injury score increased in T/HS vs. T/SS mice and was decreased in the P2X4 KO and 5-BDBD pretreated mice (Fig. 1C). Myeloid-specific P2X4 deficient mice exhibited reduced lung permeability (Fig. 2A), MPO activity (Fig. 2B) and lung injury score (Fig. 2C) compared to control mice, indicating that P2X4 receptors on myeloid cells mediate injury. Like P2X4 antagonism, selective pharmacological P2X7 blockade using A740003 reduced lung permeability (Fig. 4A), MPO activity (Fig. 4B) and lung injury score (Fig. 4C). Thus, both P2X4 and P2X7 receptors contribute to lung injury following T/HS.

### Role of P2X4 in regulating gene expression after T/HS

**Figure 3:**
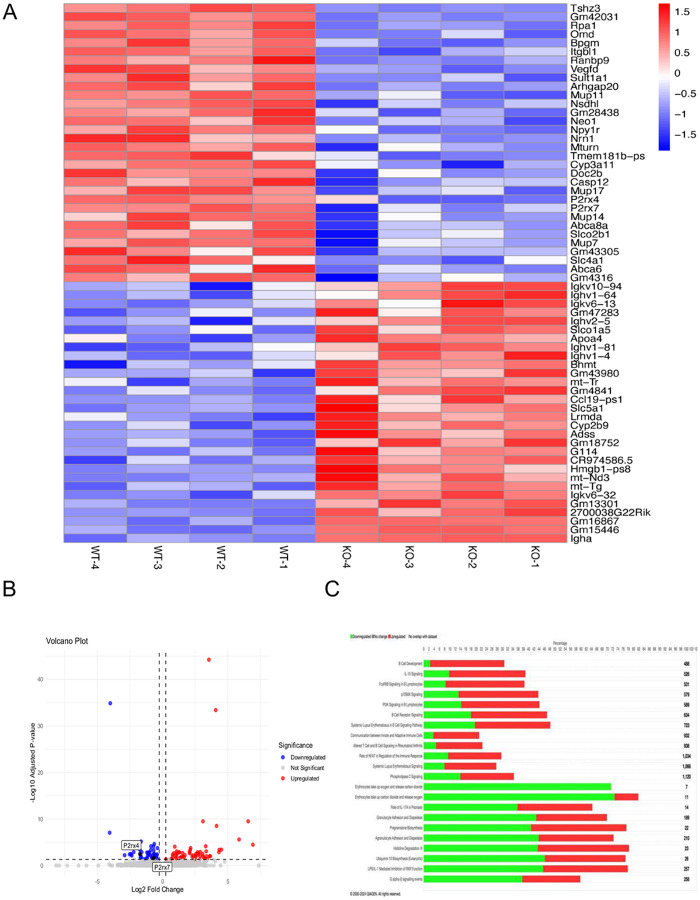
P2X4 deficiency alters gene expression in the lung of mice. RNAseq analysis was conducted on the lungs of WT and P2X4 KO mice subjected to T/HS, with four animals per group. (A) Heatmap showing the top 62 differentially expressed genes. The scale bar is log(z-score), where red colors indicate a high z-score and blue colors indicate a low z-score. (B) The volcano plot illustrates genes that are up- or down-regulated in P2X4 KO versus WT mice. (C) The stacked bar chart depicts the ratio of upregulated pathways (shown in green) and downregulated genes (denoted in red) in KO lungs as per the IPA biological function analysis. The thirty most prominent biological pathways were identified utilizing Fisher’s exact test, with a threshold of −log10 p-value > 2 (corresponding to a p-value < 0.05). The x-axis shows the percentage changes in signaling pathways.

**Figure 4:**
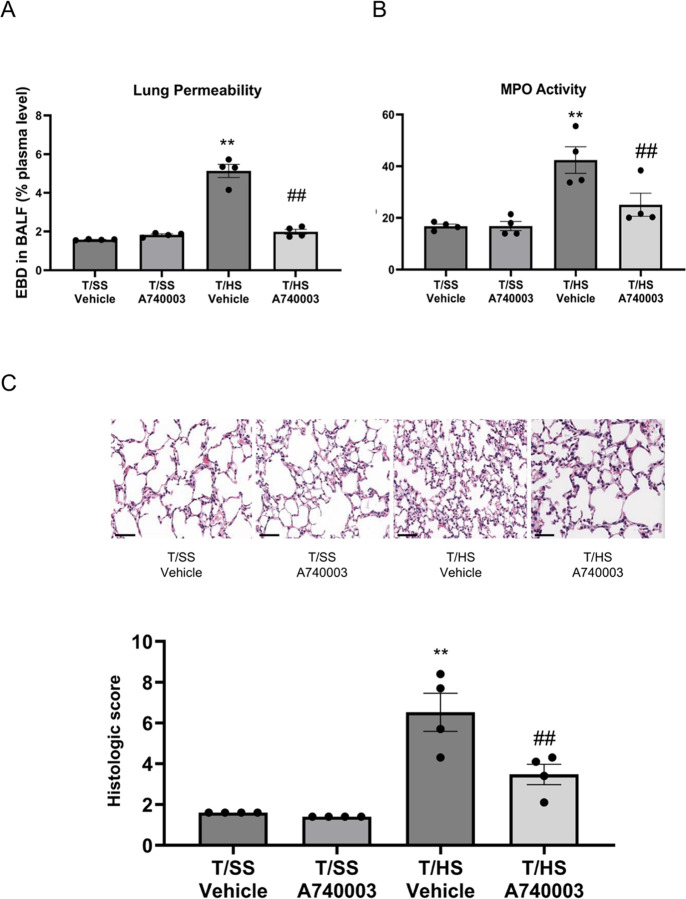
Pharmacological blockade of P2X7 receptors protects against lung injury after T/HS. Lung permeability was determined using the EBD method (A) and neutrophil sequestration was determined spectrophotometrically by measuring MPO activity (B). Histological lung injury (C) was evaluated using five independent parameters (neutrophils in the alveolar space, neutrophils in the interstitial space, hyaline membranes, proteinaceous debris filling the airspaces, and alveolar septal thickness). Data are mean ± SEM (n = 4/group). Data are mean ± SEM (n = 4/group). **p < 0.01 compared with T/SS Vehicle, ^##^p < 0.01 compared with T/HS Vehicle.

To investigate potential protective mechanisms, we conducted bulk RNAseq of the lungs from WT and P2X4 KO mice 3 hours post-T/HS. The heat map visualization shows genes that are differentially expressed between two groups. P2X7 expression was significantly reduced in P2X4 KO compared to WT mice (Fig. 3A and B). Ingenuity Pathway Analysis (Qiagen IPA software) identified significantly altered signaling pathways, including those involved in B cell development, IL-15 signaling, and cytokine signaling (Fig. 3C).

### P2X4 deficiency and blockade prevent liver and kidney injury after T/HS

**Figure 5:**
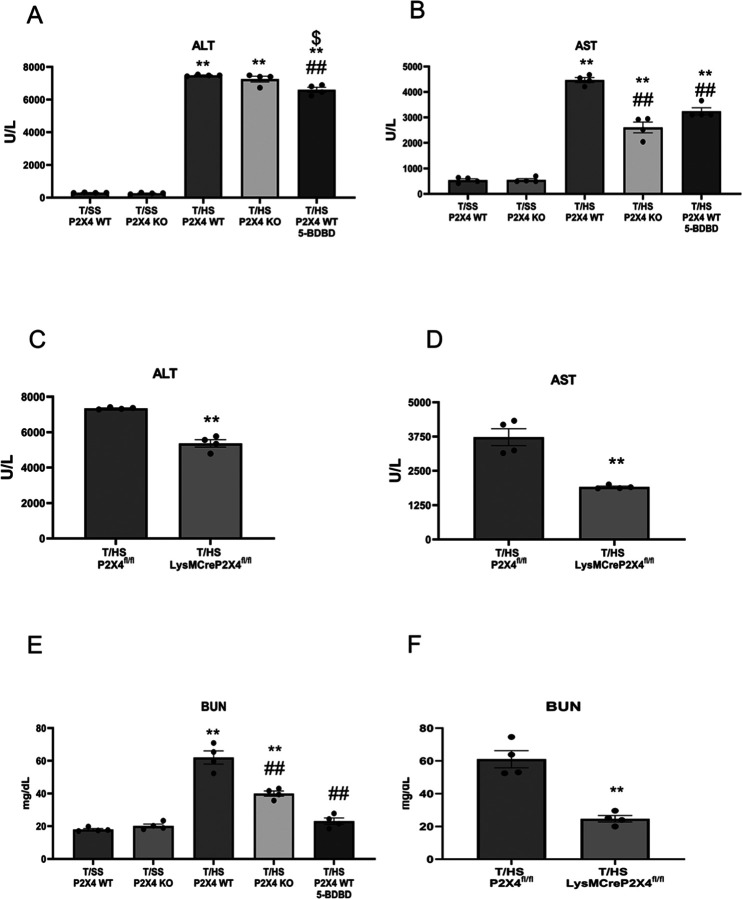
P2X4 deficiency protects liver and kidney injury after T/HS. Plasma alanine aminotransferase (ALT) (A, C) and aspartate aminotransferase (AST) (B, D) levels were used as markers of liver injury, and blood urea nitrogen (BUN) was used as a marker of kidney injury (E, F). All these markers were measured using spectrophotometry from plasma. Data are mean ± SEM (n = 4/group). **p < 0.01 compared with WT-T/SS, ^##^p < 0.05 compared with WT-T/HS, ^$^p < 0.05 compared with KO T/HS.

We then examined the role of P2X4 receptors in regulating T/HS-induced liver and kidney injury. Both P2X4 KO and 5-BDBD reduced T/HS-induced liver and kidney injury as evidenced by measuring ALT (Fig. 5A), AST (Fig. 5B) and blood urea nitrogen (Fig. 5E). We also observed that myeloid-deficient P2X4 mice had less liver (Fig. 5C and D) and kidney injury (Fig. 5F) compared to their controls.

#### P2X4 and P2X7 expression levels decrease following T/HS in vivo and LPS treatment in cultured macrophages

**Figure 6:**
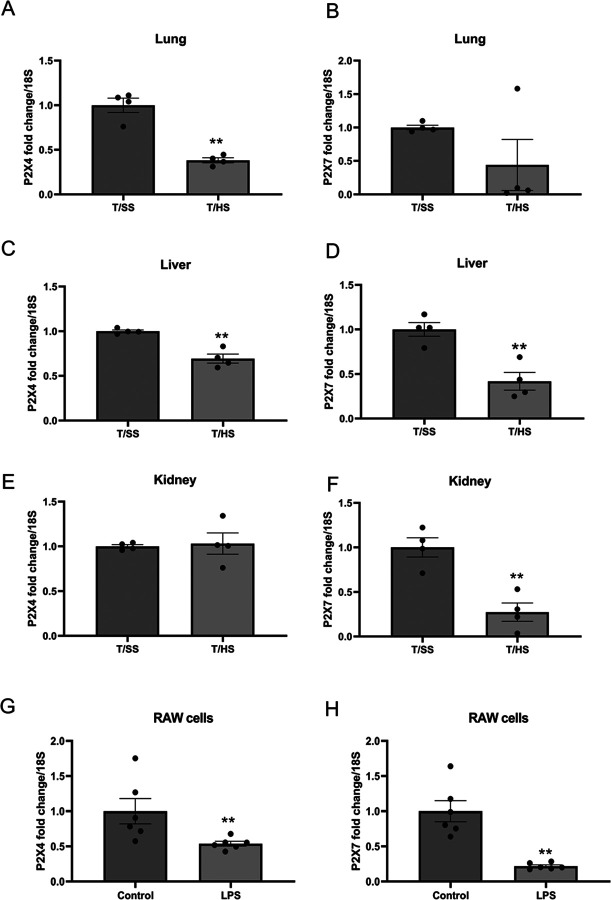
The expression of P2X4 and P2X7 is decreased in the lung, liver, and kidney of T/HS mice compared to those of T/SS. (A, B) Gene expression of P2X4 and P2X7 in lung, (C, D) liver, (E, F) and kidney of T/HS vs. T/SS mice. (G, H) Gene expression of P2X4 and P2X7 in LPS-treated RAW264.7 cells. Data are mean ± SEM (n = 4/group). **p < 0.01 compared with T/SS.

**Figure 7:**
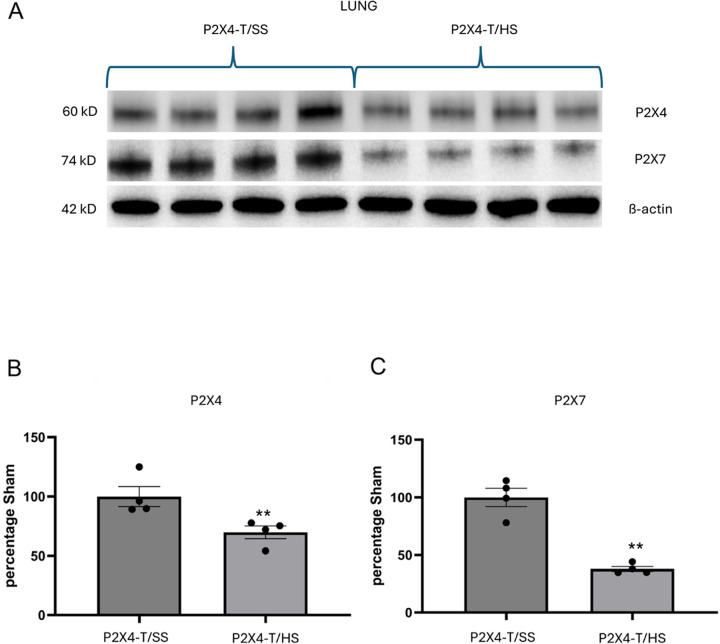
T/HS decreases P2X4 and P2X7 protein expression in lung. A is the Western blot and (B) and (C) are the quantification of P2X4 and P2X7 receptor expression expression, respectively. Data are mean ± SEM (n = 4/group). **p < 0.01 compared with T/SS.

Since we observed that the P2X7 receptor was downregulated in P2X4 KO mice, we analyzed both P2X4 and P2X7 gene and protein expression using quantitative real-time PCR and western blot in WT mice subjected to either T/SS or T/HS. We found that the mRNA transcripts of P2X4 and P2X7 were downregulated in the T/HS compared to the T/SS lung (Fig. 6A and B) and liver (Fig. 6C and D). While P2X4 was not affected by T/HS (Fig. 6E), T/HS reduced P2X7 in the kidney (Fig. 6F). Additionally, LPS treatment in RAW cells resulted in the downregulation of both P2X4 (Fig. 6G) and P2X7 mRNA (Fig. 6H). Finally, the expression of P2X4 and P2X7 proteins in lung was reduced after T/HS (Fig. 7A–C).

## Discussion

We have previously demonstrated that extracellular ATP accumulates after T/HS in mice (20). Here, we investigated the role of P2X4 and P2X7 receptors, as these two receptors are the dominant immunoregulatory P2 receptors activated by extracellular ATP (8, 21). We demonstrated that both P2X4 and P2X7 receptors contribute to the injury of lung, liver and kidney induced by T/HS in mice. These harmful effects in T/HS contrast with the protective roles of P2X4 and P2X7 receptors in sepsis (11, 22), where protection is mediated by increased bacterial killing by macrophages.

The fact that P2X4 and P2X7 receptors had similar harmful roles in T/HS aligns with the previously demonstrated similarities in the localization and function of these two receptors. Both P2X4 and P2X7 are distributed throughout the body (23). Indeed, these two receptors are widely expressed in central and peripheral neurons, microglia, and various glandular tissues such as pancreatic acinar cells and salivary glands as well as endothelial cells (24, 25). They are expressed also throughout the lung, gastrointestinal tract, liver, kidney and reproductive system (21, 26). In immune cells, P2X4 and P2X7 have been linked to similar processes, such as inflammasome activation, release of IL-1β and IL-18, production of reactive oxygen species (ROS) (13, 14), phagosome function (15, 16), autophagy, macrophage death (17), and autocrine and paracrine activation of T cells (27–29). Our data show that the expression of both receptors decreases after T/HS, and LPS downregulates the expression of both in macrophages, thereby further highlighting similarities between the two receptors. We can only speculate as to the mechanisms of this downregulation. One possibility, at least in vivo, is that endogenously released corticosteroids may be involved, as a recent study demonstrated that dexamethasone suppressed P2X4 in microglia following rat traumatic brain injury (30). However, this does not explain the in vitro effects of LPS. Clearly, detailed mechanistic studies are needed to understand the intracellular mechanisms regulating P2X4 and P2X7. Finally, in addition to highlighting similarities between the two receptors, our data may also indicate mechanistic connections. That is, it is possible that the harmful effects of P2X4 activation are mediated by P2X7, a question that will need to be addressed in further studies.

However, there are also differences between the two receptors. P2X4 is highly sensitive to ATP, being activated by nanomolar to low micromolar concentrations of extracellular ATP (31). The Ca^2+^ permeability of P2X4 is the highest among the P2X family (32). Immunoprecipitation studies have shown that P2X4 can form heteromeric assemblies with other P2X members, particularly, P2X7 (33–35). In addition, P2X4 and P2X7 are widely co-expressed particularly on immune/inflammatory cells (36).

*P2RX4* in humans is located on chromosome 12 in close proximity to *P2RX7*. This association is conserved and phylogenetic analysis shows that P2X4 and P2X7 share the same ancestral gene and were likely produced by local gene duplication (31). P2X7, however, has a 1000-fold lower affinity to ATP compared to P2X4. Thus, in murine peritoneal macrophages, exposure of the cells to low concentrations of ATP evoked a small P2X4-driven ion current, while higher ATP concentrations evoked a large P2X7-driven ion current (37). While native P2X4 appears to be predominantly localized in lysosomes in many cell types at rest (15), P2X7 is mainly found at the plasma membrane and only to some extent in intracellular compartments. Thus, it will be important to study and compare the cellular and molecular mechanisms of action of the two receptors in augmenting organ damage during T/HS.

Our data present some cues, at least with regard to P2X4. That is, using LysMcre-P2X4^fl/fl^ mice, we demonstrate that P2X4 on myeloid cells mediates the deleterious effects of P2X4 in T/HS. These results plus the fact that myeloid cells express high levels of P2X7 indicate that similar studies on the role of P2X7 receptors are warranted. The specific myeloid cell type responsible for the damaging effect of P2X4 at this stage is unknown. Future studies should focus on macrophages and neutrophils, as both cell types are targeted using the LysM driver. In contrast, dendritic cells and other granulocytes besides neutrophils, which can also have genes deleted by a LysM driver, are unlikely to be involved in T/HS.

Our study has limitations. For example, we used only male mice. While this approach is consistent with standardization of the trauma-hemorrhagic shock model and reduces variability due to estrous-related hormonal fluctuations, it limits conclusions regarding potential sex-dependent differences. Future studies will include female mice to determine whether the mechanisms identified here are conserved across sexes. Another issue concerns the timing of P2 receptor manipulation, which occurred before inducing shock in both the KO mice and the pharmacological treatment. Therefore, the therapeutic potential of targeting these receptors should be evaluated in scenarios where antagonists are administered after shock induction. Another issue is the discrepancy of our results, which indicate LPS-induced down-regulation of P2X4 mRNA in RAW264.7 macrophages and those of a previous study, which showed upregulation by LPS.

Together, these studies provide a new understanding of how ATP release and P2X4 and P2X7 receptors mediate inflammation and organ damage after T/HS, and identify potential new receptor-based therapeutic targets to improve outcomes for patients experiencing these injuries.

## Data Availability

The original data from this study will be made available upon reasonable request.
